# Massive Intradural Chondroma Masquerading as Lower Body Parkinsonism

**DOI:** 10.7759/cureus.2099

**Published:** 2018-01-22

**Authors:** Ian D Connolly, Eli Johnson, Seth Lummus, Melanie Hayden Gephart

**Affiliations:** 1 Department of Neurosurgery, Stanford University School of Medicine; 2 Department of Neurosurgery, Stanford University School of Medicine; 3 Department of Neuropathology, Stanford University School of Medicine

**Keywords:** chondroma, lower body parkinsonism, intradural chondroma, dural convexity, brain tumor, impaired gait, movement disorder

## Abstract

Intracranial chondromas of the dural convexity are exceedingly rare with less than 30 reported in the literature to date. We report a massive intradural convexity chondroma in a patient initially thought to have a frontal gait neurodegenerative disorder. This large tumor required a complex, piecemeal surgical resection due to the dense, fibrous nature of the tumor and the proximity of crucial structures. The patient had complete resolution of her preoperative symptoms after surgical excision.

## Introduction

Intracranial chondromas are benign, slow-growing neoplasms of mature hyaline cartilage. They occur intracranially in rare cases with an estimated incidence of 0.2% to 0.5% of all intracranial neoplasms [1]. Most commonly they arise from cartilage rests of the skull base in the sphenopetrosal, sphenoclival, or petroclival junctions, although they can also occur in the parasellar region, choroid plexus, falx, or the dura [1]. The dural convexity is a rare location of occurrence with less than 30 reported cases [1-2]. Unlike meningiomas, chondromas have no gender preference and typically present in patients 20 to 60 years of age [1]. Due to the slow-growing nature of this tumor, patients typically present with a long history of symptoms related to mass effect. These include seizures, increased intracerebral pressure, hydrocephalus due to CSF blockage, and cranial nerve palsies. Good long-term outcomes are achieved with complete resection. 

Here, we present a rare case of a massive intradural convexity chondroma in a patient thought to have a frontal gait neurodegenerative disorder. We also discuss the relevant literature on this rare intracranial tumor. To our knowledge, this is the first report of a dural convexity chondroma that manifested as lower body Parkinsonism (bilateral lower extremity akinesia, shuffling gait, and frequent gait freezing with no upper extremity symptoms) [3].

## Case presentation

Presentation

**Figure 1:**
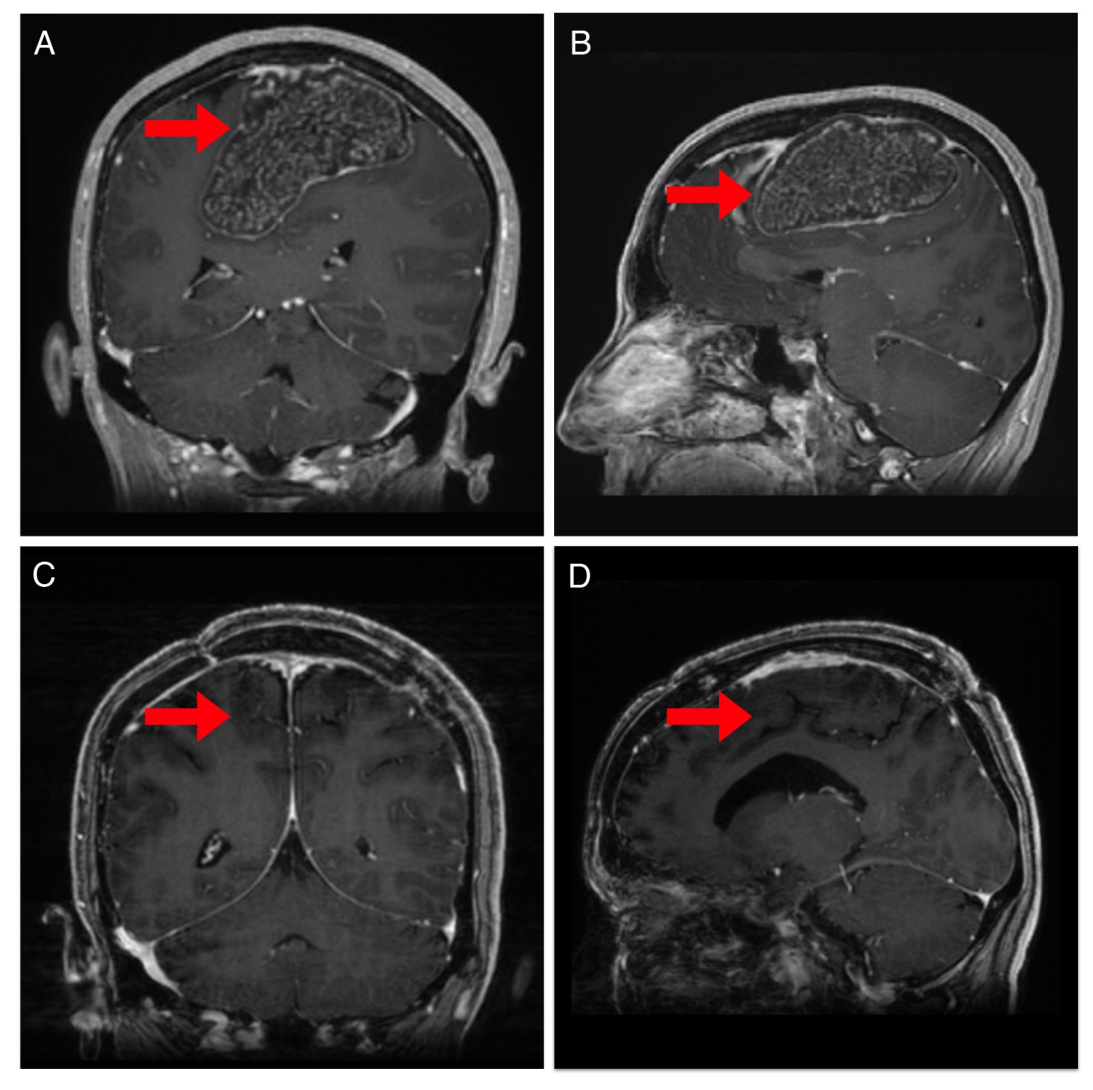
Pre and postoperative magnetic resonance imaging (MRI) Coronal (A) and sagittal (B) MRI imaging (Bravo sequence) revealed an 8.7 x 5.5 x 4.6 cm tumor (red arrow). Coronal (C) and sagittal (D) postoperative MRI imaging (Bravo sequence) showing complete resection of the tumor (red arrow).

**Figure 2:**
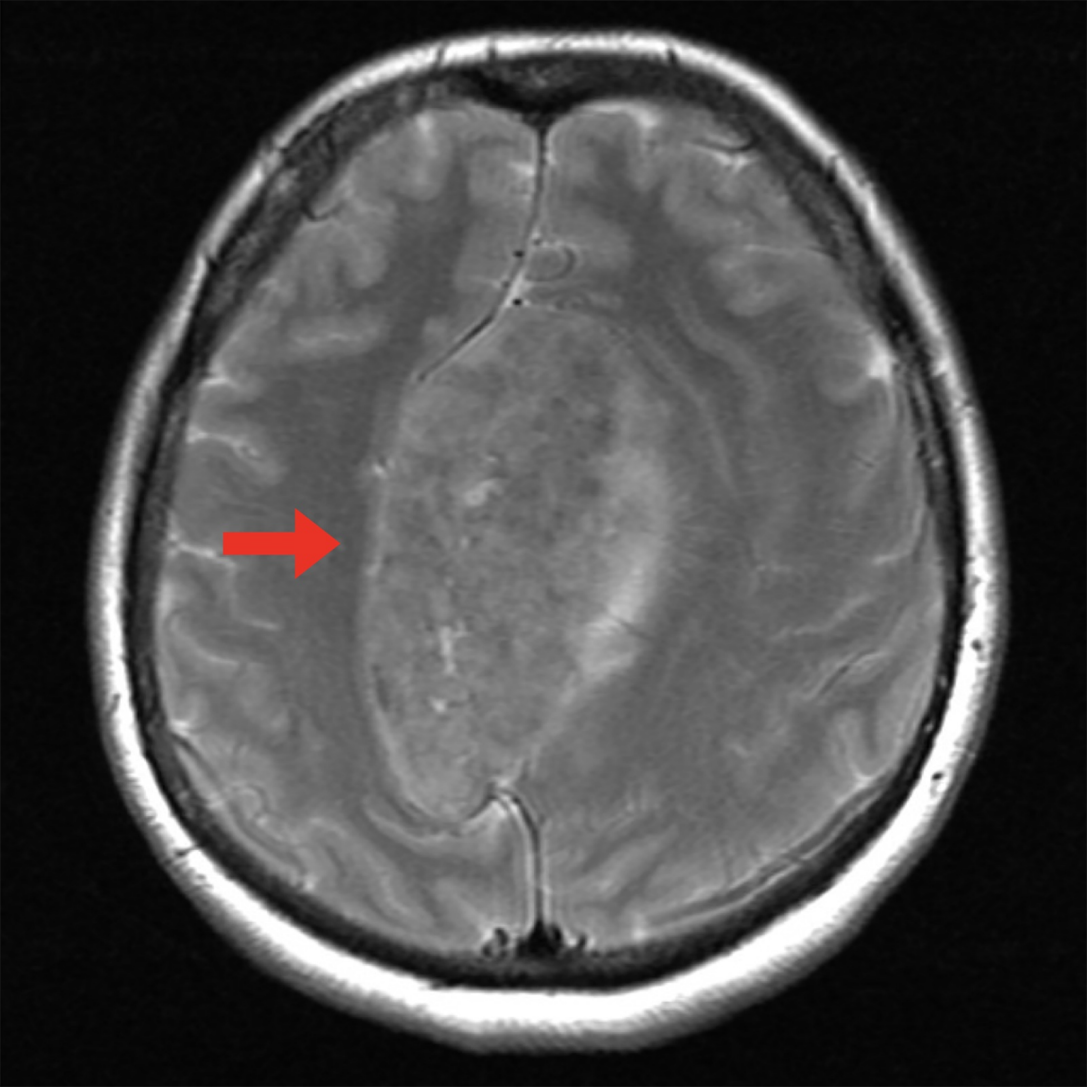
Preoperative T2-weighted magnetic resonance imaging (MRI) Axial T2-weighted MRI demonstrating lack of edema in peritumoral brain (red arrow).

A 54-year-old, right-handed female presented with progressive difficulty ambulating, worsening cognition, and left hand tremor. She had been using a front wheeled walker to ambulate for two years. At the point of presentation to our clinic, she was wheelchair-bound and unable to ambulate. She was referred to the movement disorder clinic, where her gait was described as primarily lower body Parkinsonism with akinesia, shuffling gait, and frequent gait freezing. With the exception of a small left hand tremor, she had no upper extremity symptoms. She was suspected to have a frontal gait neurodegenerative disorder possibly due to vascular parkinsonism or normal pressure hydrocephalus. As a part of her initial workup, she received a magnetic resonance imaging (MRI) scan with contrast, which showed a large (8.5 x 5.5 x 4.5 cm), heterogeneously contrast-enhancing lesion (Figure 1A, 1B). This lesion compressed the superior sagittal sinus (SSS), displaced the bilateral anterior cerebral arteries (ACA), created severe brain compression, and was responsible for her bilateral lower extremity weakness. T2-weighted imaging showed minimal edema (Figure 2). An atypical meningioma was initially suspected, and the patient elected to proceed with surgical resection of the mass after the risks, benefits, and alternatives to surgery were discussed.

Surgery

**Figure 3:**
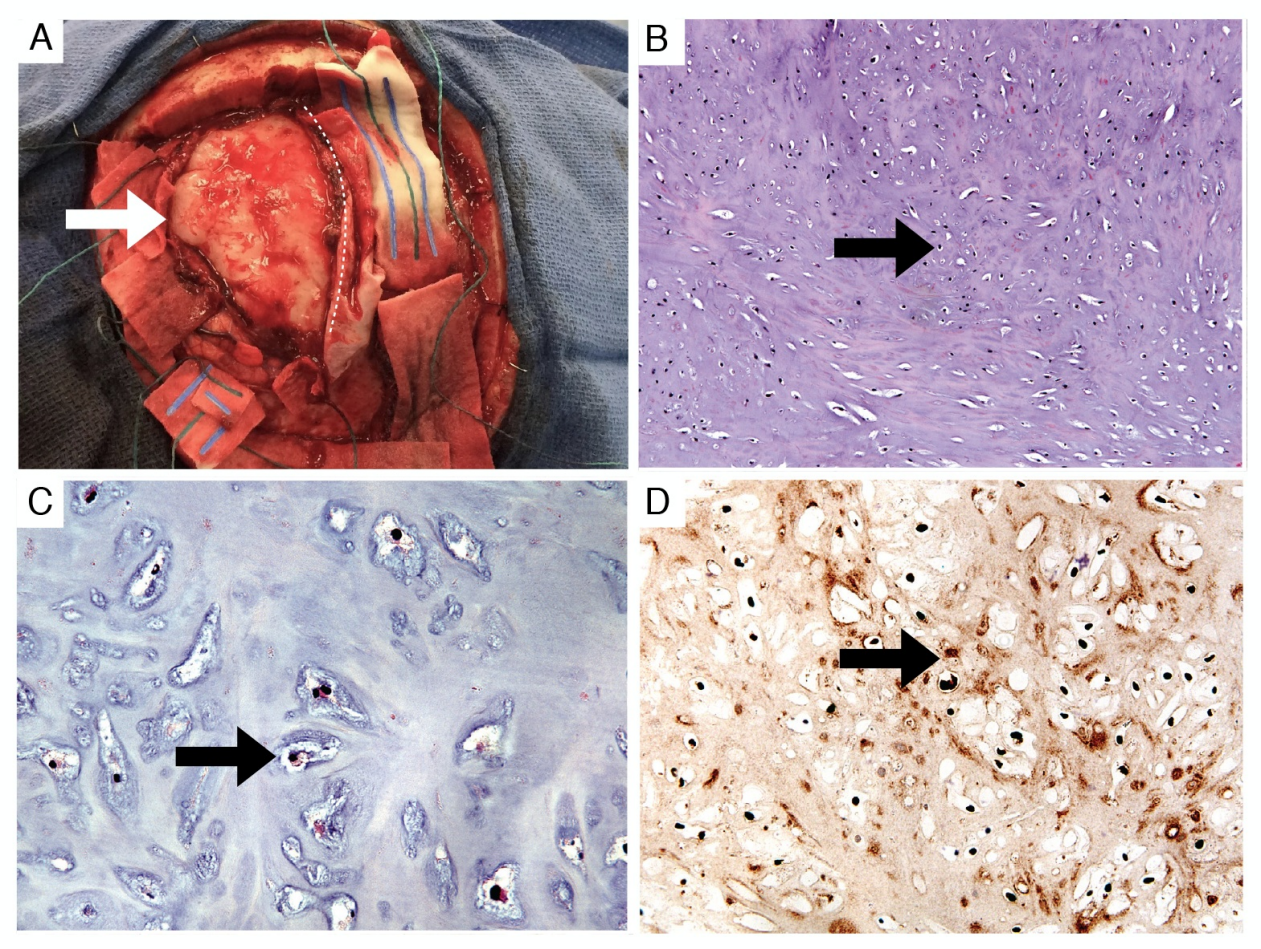
Gross appearance and histology of the tumor Appearance of the mass (white arrow) during piecemeal resection (A). The superior sagittal sinus is protected under a midline patty (dotted line). Routine histologic sections demonstrated cytologically bland chondrocytes (B) 100x and (C) 400x H&E (black arrows). Immunohistochemistry for S100 protein was positive, confirming a chondrocyte origin (D) 200x, (black arrow). H&E: hematoxylin and eosin

A trapdoor incision was made on the patient's left side and crossed the midline. This was necessary given the large extent of the tumor, including to the contralateral side past the SSS (Figure 3A). Prior to the dural opening, the extremely fibrous mass could readily be palpated under the dura. Opening the dura revealed a white, cartilaginous-appearing mass (Figure 3A). Resecting the mass was complex given the very large size, the calcified and fibrous nature of the mass, the underlying and severely compressed primary motor cortex and tracks, and the displacement and involvement of the bilateral ACA arteries and multiple crucial draining veins. The large lesion displaced but did not invade the falx cerebri and surrounding bridging veins. Multiple instruments were attempted to dissociate the mass for piecemeal extraction. The surrounding vasculature was meticulously and completely dissected off the tumor capsule, preserving their structural integrity and function. The postoperative course was unremarkable and the patient was discharged three days after surgery. Routine postsurgical MRI at three months revealed gross total resection (Figure 1C, 1D). The patient had rapid recovery of her motor function, was able to ambulate independently within weeks, and had a complete neurologic recovery at one month. At her three month follow-up, her preoperative neurologic symptoms had resolved, and she had no postoperative complications.

Histopathology

Gross inspection revealed a firm-to-hard, white, homogenous, smooth-surfaced tissue. Histologic sections showed a homogeneous, cytologically-bland cartilaginous neoplasm. No mitoses were appreciated. Occasional binucleated chondrocytes and rare chondrocytes with larger nuclei were seen (Figure 3B, 3C, 3D). The gross, histologic, and immunohistochemical findings were consistent with those of a benign chondroma arising from the dura.

## Discussion

The pathogenesis of intracranial chondromas remains controversial. Proposed theories include metaplasia of meningeal fibroblasts or perivascular mesenchymal cells, the growth of heterotrophic chondrocytes that develop in the meninges during early ontogenesis, or the cartilaginous activation of meningeal connective tissue after trauma or inflammation [2].

Similar to meningiomas, intradural chondromas appear dural-based, extramedullary, and well-circumscribed masses. Although rare, they differ from a meningioma on imaging by the absence of a dural tail and heterogeneous contrast enhancement [1]. Depending on the degree of calcification, they can also appear as hyper or hypodense [1], which is also true of meningiomas. In some cases, they may contain a necrotic hypodense center [1]. On MRI imaging, chondromas appear as iso to hypodense on T1-weighted images and mixed iso to hyperdense on T2-weighted images [1-2].

It is important to distinguish chondromas from other connective tissue tumors that can involve the CNS, such as chordomas and chondrosarcomas that display similar MRI imaging features (hypodense on T1 and hyperdense on T2 MRI imaging) [4]. Chordomas arise from notochord remnants and are usually found in the midline [4]. Chondrosarcomas also tend to occur at the midline, though they can sometimes stray laterally [4]. Histologically, malignant features, such as hypercellularity, cellular atypia and increased mitotic activity, and nuclei per lacuna, are more suggestive of a chondrosarcoma. In the absence of these features, aggressive and destructive features on imaging favor a low-grade chondrosarcoma.

Less than 30 of these rare clinical entities have been reported in the literature. The most common presentations in previously reported cases of dural convexity chondromas tend to be headaches and seizures. Based on our review of previous cases of dural convexity chondromas presented by Abeloos, et al., we were only able to identify two likely instances of presentations with impaired ambulation [5-6]. Hardy, et al. described a patient who experienced progressive weakness of the right arm and leg over the course of six months, along with headache and intermittent dizziness [5]. Berkmann and colleagues reported a patient presenting with headaches and left-sided pain and weakness causing the patient to become bedridden [6]. Our case is unique in that the patient presented with gait difficulty that was out of proportion to her mild weakness. To our knowledge, our case is the first to present with movement difficulties that specifically manifested as lower body Parkinsonism.

If small and asymptomatic, intradural chondromas can be observed for interval growth with subsequent MRI brain imaging. Although the circumscribed nature of most intracranial chondromas facilitates surgical resection, as the tumors grow they frequently engulf surrounding brain vasculature that must be preserved. In addition, the large size and fibrous nature of these lesions complicate their surgical resection, particularly when adjacent to the eloquent cortex, as in the case of our patient. Following maximal safe surgical resection, postoperative MRI brain should be obtained at three months as a new baseline. Repeat MRIs would be at the discretion of the treating physician, and we have elected for yearly scans for the first couple years. Radiation therapy is not recommended in cases of residual tumor or inoperable tumors as it is thought that this increases the risk of malignant transformation [1].

## Conclusions

Dural convexity chondromas are rare, histologically benign intracranial lesions. Intracranial chondromas may be difficult to distinguish radiographically from meningiomas but generally lack a dural tail and are heterogeneously enhancing. Although surgically curable, these lesions present technical challenges, given that they are frequently large at the time of presentation and involve the eloquent cortex and crucial vascular anatomy.
